# Supplementary material for: Proteomic profiling reveals diagnostic signatures and pathogenic insights in multisystem inflammatory syndrome in children
Source: Commun Biol. 2024 Jun 5;7:688. doi: 10.1038/s42003-024-06370-8 (PMC11153518; doi:10.1038/s42003-024-06370-8)
Supplement: Supplementary file 2 — Description of Additional Supplementary Files [file 42003_2024_6370_MOESM2_ESM.pdf]

Description of Additional Supplementary Files

File name: Supplementary Data 1

Description: The final dataset behind the graphs in the paper.
