# Supplementary material for: Proteomic profiling reveals diagnostic signatures and pathogenic insights in multisystem inflammatory syndrome in children
Source: Commun Biol. 2024 Jun 5;7:688. doi: 10.1038/s42003-024-06370-8 (PMC11153518; doi:10.1038/s42003-024-06370-8)
Supplement: Supplementary file 7 — Reporting summary [file 42003_2024_6370_MOESM7_ESM.pdf]

nature portfolio

Corresponding author(s): Ulrikka Nygaard

Last updated by author(s): Apr 25, 2024

Reporting Summary

Nature Portfolio wishes to improve the reproducibility of the work that we publish. This form provides structure for consistency and transparency in reporting. For further information on Nature Portfolio policies, see our Editorial Policies and the Editorial Policy Checklist.

Statistics

For all statistical analyses, confirm that the following items are present in the figure legend, table legend, main text, or Methods section.

n/a	Confirmed	
☐	☒	The exact sample size (*n*) for each experimental group/condition, given as a discrete number and unit of measurement
☐	☒	A statement on whether measurements were taken from distinct samples or whether the same sample was measured repeatedly
☐	☒	The statistical test(s) used AND whether they are one- or two-sided<br>*Only common tests should be described solely by name; describe more complex techniques in the Methods section.*
☐	☒	A description of all covariates tested
☐	☒	A description of any assumptions or corrections, such as tests of normality and adjustment for multiple comparisons
☐	☒	A full description of the statistical parameters including central tendency (e.g. means) or other basic estimates (e.g. regression coefficient) AND variation (e.g. standard deviation) or associated estimates of uncertainty (e.g. confidence intervals)
☐	☒	For null hypothesis testing, the test statistic (e.g. *F*, *t*, *r*) with confidence intervals, effect sizes, degrees of freedom and *P* value noted<br>*Give P values as exact values whenever suitable.*
☒	☐	For Bayesian analysis, information on the choice of priors and Markov chain Monte Carlo settings
☐	☒	For hierarchical and complex designs, identification of the appropriate level for tests and full reporting of outcomes
☒	☐	Estimates of effect sizes (e.g. Cohen's *d*, Pearson's *r*), indicating how they were calculated

Our web collection on statistics for biologists contains articles on many of the points above.

Software and code

Policy information about availability of computer code

Data collection	Spectronaut version 17 was used to analyze the MS .raw files
Data analysis	Data analysis was carried out in a Jupyter Notebook available at github: https://github.com/annelaura/MIS-C

For manuscripts utilizing custom algorithms or software that are central to the research but not yet described in published literature, software must be made available to editors and reviewers. We strongly encourage code deposition in a community repository (e.g. GitHub). See the Nature Portfolio guidelines for submitting code & software for further information.

Data

Policy information about availability of data

All manuscripts must include a data availability statement. This statement should provide the following information, where applicable:

- Accession codes, unique identifiers, or web links for publicly available datasets
- A description of any restrictions on data availability
- For clinical datasets or third party data, please ensure that the statement adheres to our policy

The mass spectrometry proteomics data have been deposited to the ProteomeXchange Consortium via the Proteomics Identifications Database (PRIDE) partner repository,21 with the dataset identifier PXD045661.

Research involving human participants, their data, or biological material

Policy information about studies with human participants or human data. See also policy information about sex, gender (identity/presentation), and sexual orientation and race, ethnicity and racism.

Reporting on sex and gender	We used the term 'sex', obtained from the social security numbers. No sex-based analyses were performed.
Reporting on race, ethnicity, or other socially relevant groupings	Data on race, ethnicity or other socially relevant groupings were not available.
Population characteristics	Patients aged (range) 1-15 years with multisystem inflammatory syndrome in children (MIS-C, n=27) and controls with viral (n=22) and bacterial infections (n=28), Kawasaki (n=7) and severe sepsis (n=10), recruited from all the Danish pediatric departments. Clinical characteristics including hypotension, cardiac, gastrointestinal, hematologic, dermatologic, neurologic, respiratory and renal involvement. CRP maximum range 0-402 mg/L. Treatment including antibiotic therapy, inotropes, immunoglobulins, glucocorticoids and anakinra. Intensive care unit stay. Length of hospital stay range 0-21 days.
Recruitment	The patients were recruited from all 18 pediatric hospital departments in Denmark. However, the majority was recruited from one of the 4 referral pediatric hospital departments.
Ethics oversight	The Ethics Committee of the Capital Region of Denmark (H-20028631). The Danish Data Protection Agency (P-2019-29).

Note that full information on the approval of the study protocol must also be provided in the manuscript.

Field-specific reporting

Please select the one below that is the best fit for your research. If you are not sure, read the appropriate sections before making your selection.

☒ Life sciences ☐ Behavioural & social sciences ☐ Ecological, evolutionary & environmental sciences

For a reference copy of the document with all sections, see nature.com/documents/nr-reporting-summary-flat.pdf

Life sciences study design

All studies must disclose on these points even when the disclosure is negative.

Sample size	Since the study is explorative in nature, sample size determination was not applicable. However, in preliminary studies of bacterial and viral infections, 15-20 patients with a specific infection were sufficient to establish a signature with sensitivity and specificity >90%. Thus, we included at least 20 patients with MIS-C and febrile controls based on the magnitude encountered in these studies.
Data exclusions	Three samples were excluded due to low numbers of measurable proteins, all from children with sepsis.
Replication	We conducted analyses on both an internal (Danish) and an external (US) validation cohort.
Randomization	Randomization was not relevant because the study is not a randomized controlled trial.
Blinding	Blinding was not relevant because the study is not a randomized controlled trial.

Reporting for specific materials, systems and methods

We require information from authors about some types of materials, experimental systems and methods used in many studies. Here, indicate whether each material, system or method listed is relevant to your study. If you are not sure if a list item applies to your research, read the appropriate section before selecting a response.

Materials & experimental systems

n/a	Involved in the study	
☒	☐	Antibodies
☒	☐	Eukaryotic cell lines
☒	☐	Palaeontology and archaeology
☒	☐	Animals and other organisms
☐	☒	Clinical data
☒	☐	Dual use research of concern
☒	☐	Plants

Methods

n/a	Involved in the study	
☒	☐	ChIP-seq
☒	☐	Flow cytometry
☒	☐	MRI-based neuroimaging

Clinical data

Policy information about clinical studies

All manuscripts should comply with the ICMJE guidelines for publication of clinical research and a completed CONSORT checklist must be included with all submissions.

Clinical trial registration	NCT05334134
Study protocol	The study protocol is available within the clinical trial registration.
Data collection	The patients were recruited from all Danish pediatric departments from April 1, 2020, to March 15, 2022. Data included clinical information and venous blood samples.
Outcomes	Primary: Proteomic signature. Secondary: Disease pathogenesis.

Plants

Seed stocks	No plant specimens were collected.
Novel plant genotypes	No plant specimens were collected.
Authentication	No plant specimens were collected.
